# Cooperation, Defection, and Collapse: A Multiscale Game Theory Framework for Emphysema Progression

**DOI:** 10.3390/cells15161470

**Published:** 2026-08-17

**Authors:** Jerome Cantor

**Affiliations:** School of Pharmacy and Applied Health Sciences, St John’s University, Queens, NY 11439, USA; cantorj@stjohns.edu

**Keywords:** game theory, pulmonary emphysema, percolation theory, extracellular matrix crosslinking, combined pulmonary fibrosis and emphysema

## Abstract

In the current paper, pulmonary emphysema is hypothesized to emerge from a nonlinear breakdown of cooperation across two tightly coupled systems: the extracellular matrix (ECM) crosslink network and the cellular populations responsible for its maintenance. To formalize this concept, we construct a game-theoretic model that unifies the mechanical failure, inflammatory changes, and percolation-driven tissue collapse that are recognized features of the disease. At the ECM level, elastin and collagen crosslinks are modeled as players in an iterated Prisoner’s Dilemma, where cooperation corresponds to maintaining structural integrity, and defection corresponds to rupture under mechanical stress. At the cellular level, fibroblasts, macrophages, and neutrophils engage in a parallel strategic game in which repair reflects cooperative activity, and protease- or oxidant-producing phenotypes are indicative of defection. These parallel games are coupled through bidirectional payoff modulation, generating a dynamical system with bistability, tipping points, and runaway positive feedback. As the fraction of intact crosslinks falls below a critical percolation threshold, global network connectivity collapses and lung function drops precipitously. This framework explains the characteristic features of pulmonary emphysema, including spatial heterogeneity, abrupt acceleration, and irreversibility as emergent properties of coupled cooperation–defection dynamics, and identifies new leverage points for stabilizing cooperation and preventing catastrophic network failure in early disease. In support of this hypothesis, we present previously published studies from our laboratory involving measurements of elastin-specific desmosine crosslinks in human postmortem emphysematous lungs showing a marked increase in tissue crosslink density at the early stage of the disease, and accelerating loss of these crosslinks as airspace enlargement progresses, consistent with initial cooperation followed by defection. This conceptual framework is then applied to the poorly understood lung disease, Combined Pulmonary Fibrosis and Emphysema, to provide a potential mechanism for its pathogenesis.

## 1. Introduction

**Figure 1:**
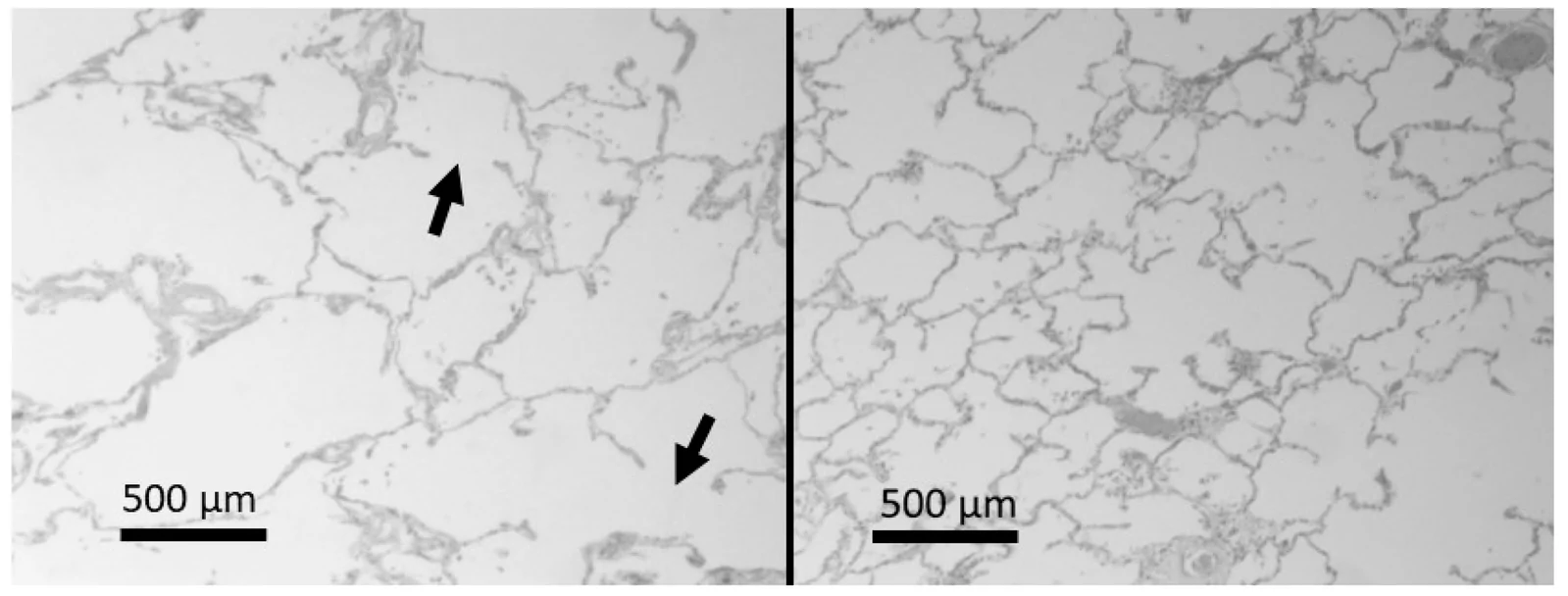
(**Left**) Photomicrograph of emphysematous human lung showing enlarged airspaces with ruptured alveolar walls (arrows). (**Right**) Photomicrograph of normal human lung for comparison. Reprinted with permission [8].

Pulmonary emphysema is characterized by the progressive and irreversible destruction of alveolar tissue, leading to airspace enlargement and decreased gas exchange [Figure 1] [1,2]. Despite decades of research into its molecular underpinnings, emphysema continues to resist the development of disease-modifying therapies and therapeutic intervention is largely limited to symptomatic relief of airway inflammation [3]. Current understanding of the pathogenesis of the disease emphasizes the central role of proteolytic enzymes, particularly matrix metalloproteinases (MMPs) and neutrophil elastase, in degrading the structural scaffold of the lung, which may be facilitated by the effects of oxidative stress in inactivating endogenous protease inhibitors [4,5]. However, this mechanistic account, while accurate at the molecular level, does not fully explain some of the more puzzling clinical features of emphysema, including the long latency period followed by sudden acceleration of decline, the pronounced spatial heterogeneity of tissue destruction within an individual lung, and the irreversibility of established disease even after removal of the causative agent, such as cigarette smoke [6,7].

These features suggest that emphysema is not merely a matter of quantitative excess in destructive molecular activity but reflects a qualitative shift in the organizational state of lung tissue, a transition from a regime of maintained cooperation among structural and cellular elements to one of self-reinforcing collapse. Disease progression is not uniform across individuals: some smokers show a rapid, accelerating decline while others follow trajectories indistinguishable from non-smokers [9,10]. Standard models of proportional damage accumulation cannot account for this bimodality without invoking threshold effects and bifurcation dynamics.

Game theory, the mathematical discipline concerned with strategic interaction among agents whose payoffs depend on the actions of others, provides a rigorous framework for modeling such threshold behavior [11,12]. Originally developed in economics and subsequently applied across evolutionary biology, ecology, immunology, and social science [13,14,15], game theory has proven remarkably effective at explaining how cooperative and defective behaviors arise, persist, or collapse in complex systems. The Prisoner’s Dilemma, in particular, has become the canonical model for the breakdown of cooperation under conflicting incentives and has been applied to everything from microbial biofilms to tumor evolution [16,17].

The present paper proposes a multiscale game-theoretic model of emphysema progression. We model structural crosslinks in the extracellular matrix (ECM) as agents playing an iterated Prisoner’s Dilemma, where cooperation means maintaining mechanical integrity, and defection means rupturing under mechanical load. Critical cell populations, fibroblasts, macrophages, and neutrophils, are modeled as agents in a parallel game, where cooperative phenotypes are repair-oriented and defective phenotypes produce proteases and oxidants that degrade ECM. These two games are bidirectionally coupled through payoff modulation, creating a dynamical system that exhibits bistability and tipping-point behavior consistent with the clinical course of emphysema [18,19].

This work is distinct from, and extends, our previous publications on emphysema pathogenesis reviewed biochemical and cellular evidence for extracellular matrix crosslink turnover and cellular phenotype switching in emphysema without proposing a formal game-theoretic or percolation-theoretic mechanism [20,21,22]. The present manuscript’s novel contributions relative to this prior work are threefold: (i) it couples the cellular and extracellular matrix games explicitly to a percolation model of network connectivity, producing a single bistable dynamical system with an explicit, quantifiable tipping point rather than two independently described games; (ii) it relates the resulting tipping-point prediction to quantitative postmortem human crosslink data, identifying an empirically measurable approximation of the percolation threshold, p(c); and (iii) it extends the coupled framework to a three-strategy game and a dual-threshold percolation model to explain combined pulmonary fibrosis and emphysema.

## 2. Background and Biological Context

### 2.1. The Architecture of Lung Structural Integrity

The mechanical resilience of lung tissue depends on the integrated behavior of the extracellular matrix, a fibrous scaffold composed primarily of type I and III collagen and elastin [23]. Collagen fibers provide tensile strength, resisting deformation under load, while elastin confers elastic recoil, the capacity to return to resting geometry after inflation [24,25]. Both structural proteins are extensively crosslinked through covalent bonds catalyzed by lysyl oxidase, an enzyme whose activity is required for proper ECM assembly and whose impairment, whether by genetic mutation, copper deficiency, or oxidative inactivation, produces structurally vulnerable networks susceptible to mechanical failure [26,27].

The mechanical behavior of such a fibrous network cannot be understood by examining individual fibers in isolation. Network properties such as rigidity, strength, and the critical load required to initiate cascading failure are emergent properties of connectivity [28]. Percolation theory, a branch of statistical physics, provides quantitative tools for analyzing these connectivity properties [29,30]. In a percolation model, a network transitions abruptly from a globally connected state to a fragmented one when the fraction of intact bonds falls below a critical threshold, pc. Near this threshold, the mechanical response of the network changes qualitatively, and the system no longer distributes load effectively across many elements, local stress concentrations form, and failure of individual elements accelerates failure of neighbors [31,32,33].

**Figure 2:**
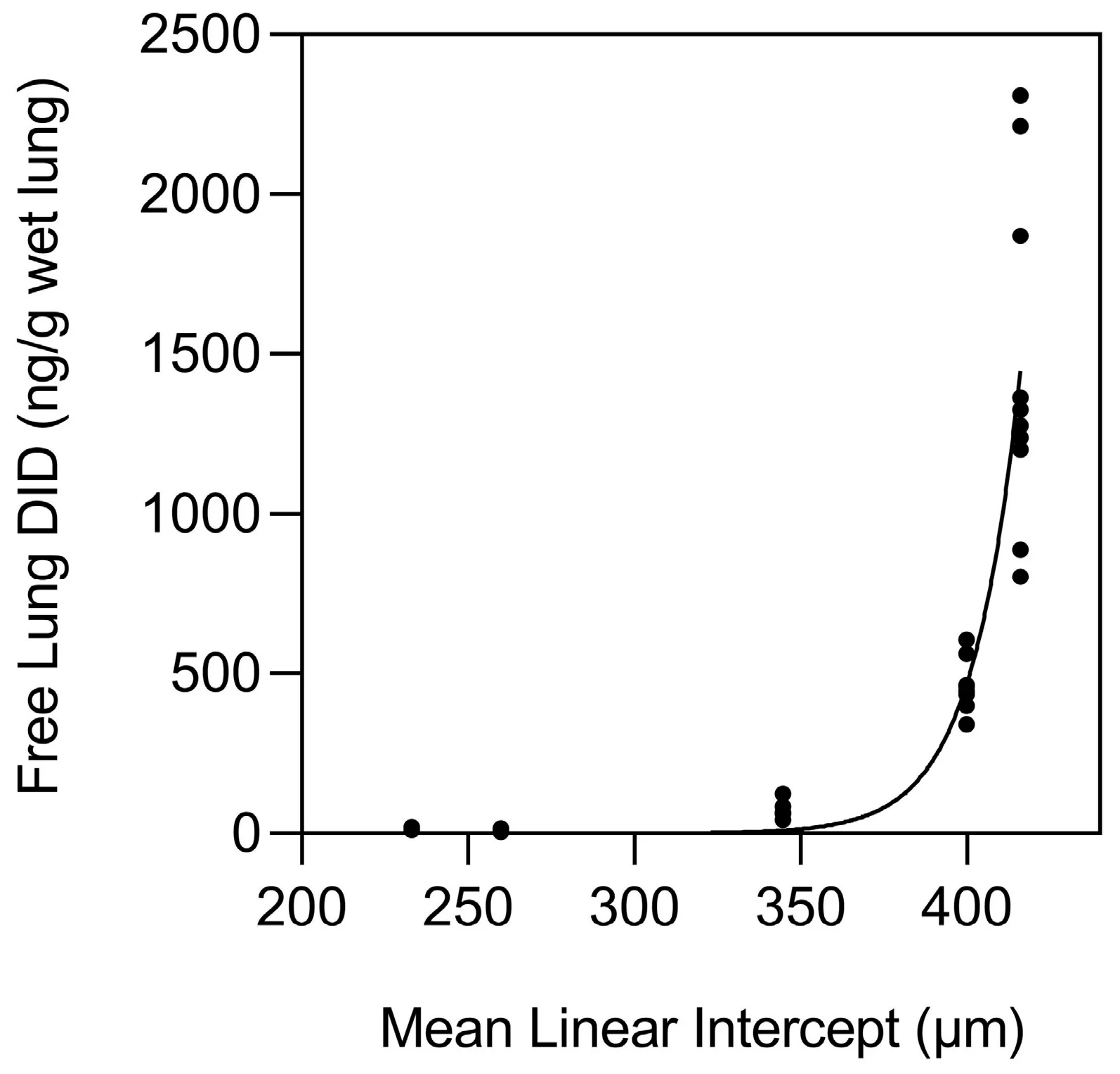
The levels of peptide-free desmosine and isodesmosine (DID) in human postmortem emphysematous lungs increase exponentially as alveolar diameter approaches 400 µm. This finding reflects the sudden collapse of alveolar wall structural integrity as the cooperative crosslink fraction approaches a critical threshold and defection becomes the dominant process. Reprinted with permission [8].

Recent quantitative studies of human lung tissue provide direct molecular evidence for this nonlinear pattern at the level of individual elastin crosslinks. Fagiola et al. measured free desmosine and isodesmosine (DID), elastin-specific amino acid crosslinks released upon elastic fiber breakdown, in formalin-fixed specimens spanning normal, mild-to-moderate, and moderate emphysema, and correlated these values with mean linear intercept (MLI), a morphometric index of alveolar diameter [8]. Free lung DID showed a nonlinear exponential relationship with MLI (r^2^ = 0.86 for an exponential growth model versus 0.51 for linear regression), with elastin breakdown accelerating sharply once alveolar diameter exceeded approximately 400 µm [Figure 2]. This pattern is precisely what the percolation-game framework predicts: modest, recoverable crosslink loss during the latent phase, followed by an abrupt, self-reinforcing cascade of elastin fragmentation once the network approaches its critical connectivity threshold.

**Figure 3:**
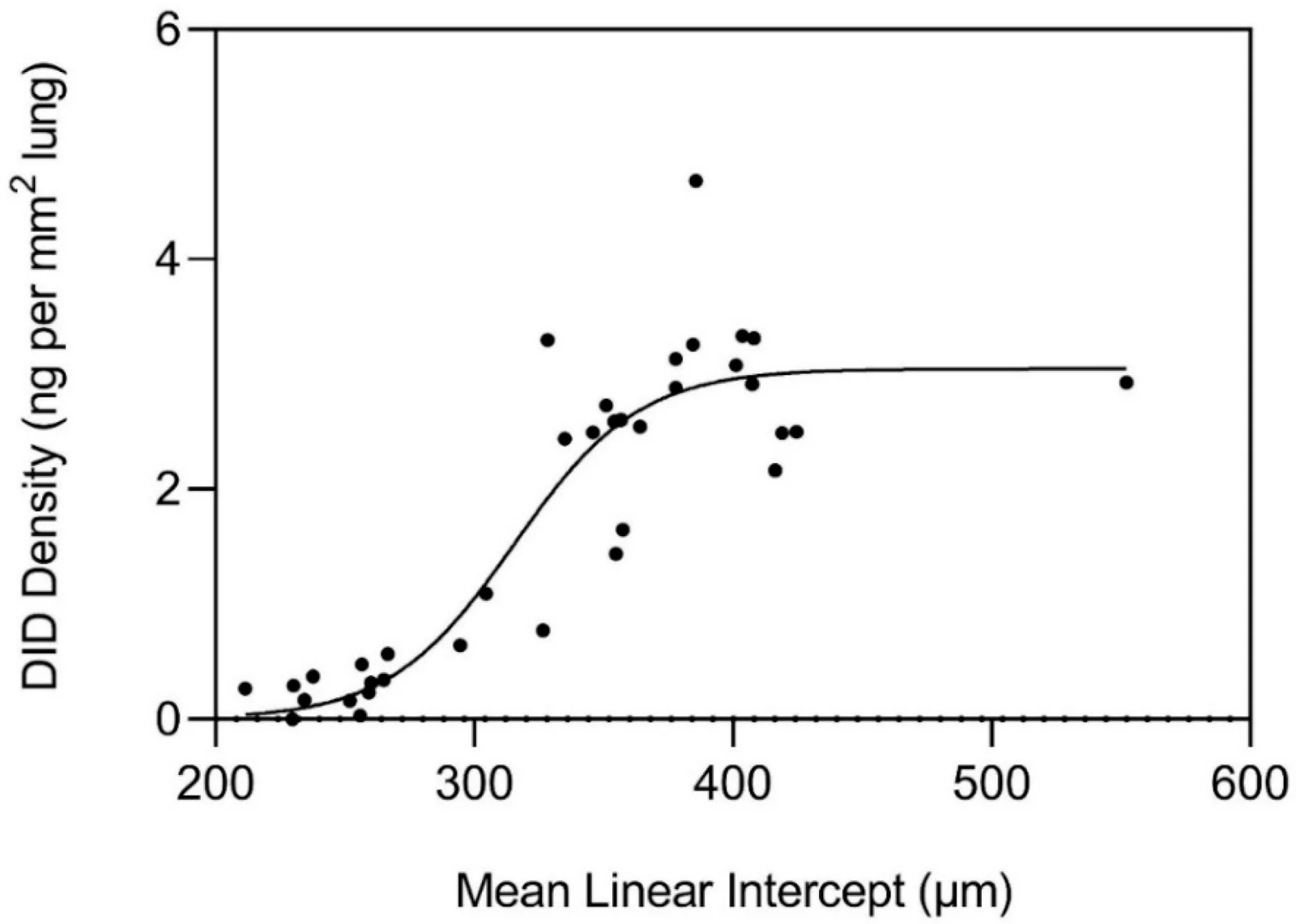
A sigmoidal increase in DID density was seen in human postmortem emphysematous lungs, consistent with a cooperative phase in crosslink formation followed by defection, as airspace size exceeds 400 µm. This transition coincides with the exponential increase in crosslink release from elastic fibers (Figure 2). Reprinted with permission [8].

Complementing the free DID data, the same study measured total DID density in formalin-fixed, paraffin-embedded tissue sections as a function of MLI, capturing not only free crosslinks released from degraded fibers but also those incorporated into intact or remodeling elastic fibers. DID density showed a sigmoid (plateau-followed-by-one-phase-association) relationship with MLI (r^2^ = 0.82), rising steeply between 200 and 300 µm and leveling off near 400 µm [Figure 3]. This saturation profile reflects the compensatory proliferation of crosslinks in response to early airspace enlargement, an attempt by the tissue to maintain structural integrity through increased elastin remodeling, followed by a decompensation phase in which the repair capacity is overwhelmed, and elastin breakdown predominates. Elastic fiber surface area followed an analogous bell-shaped curve peaking near 400 µm. However, the increment in DID density far exceeded the increment in fiber area, indicating that crosslink density per fiber, rather than total fiber content, drives the early compensatory response. Together, these findings provide empirical grounding for the payoff structure, where cooperation (crosslink maintenance and proliferation) is initially upregulated in response to mechanical strain but, above a critical airspace size, transitions irreversibly to the defection attractor as the temptation payoff for defection (crosslink rupture under load) exceeds the reward for cooperation.

We acknowledge that the exponential rise in free DID and the sigmoidal (saturating) rise in DID density are, in isolation, also consistent with simpler alternative models, including plain autocatalytic (self-accelerating) proteolysis or logistic damage accumulation, neither of which requires an underlying game-theoretic or percolation mechanism. The Fagiola et al. data should therefore be read as consistent with, rather than as proof of, the percolation-game framework. Nevertheless, two features of the data offer some discriminating power. First, the coincidence of the inflection points of the exponential (breakdown) and sigmoidal (compensatory) curves at the same approximately 400 µm MLI value is a specific quantitative prediction of a single shared threshold process acting on the same network, whereas autocatalysis and logistic damage models fit each curve independently and carry no structural requirement that their inflection points coincide. Second, the percolation account predicts that the sharpness of the transition should depend on network connectivity parameters that are independently measurable by imaging-based structural analysis, a prediction that a purely kinetic autocatalysis model does not make. Directly discriminating among these alternatives will require fitting all three model classes to the same dataset with formal model-comparison statistics, which is a priority for future quantitative work based on the formalization in Section 3.4.

### 2.2. The Role of the Extracellular Matrix in Lung Homeostasis

The ECM of the lung is not a passive scaffold but a dynamic signaling environment whose biochemical state actively regulates cellular behavior [34]. Proteolytic fragments of ECM proteins, termed matrikines, bind cell-surface receptors and modulate inflammatory, proliferative, and apoptotic programs in fibroblasts, macrophages, and epithelial cells [35,36]. Among the best characterized are the elastin-derived peptides (EDPs), which signal through the elastin-binding protein (EBP) and activate macrophage secretion of MMP-12 and pro-inflammatory cytokines [37].

Collagen crosslinking density is a principal determinant of tissue mechanical integrity and is regulated by the balance of lysyl oxidase activity, promoting crosslink formation, and MMP activity, which degrades crosslinked fibrils [38,39,40]. In cigarette smokers, reactive oxygen species (ROS) generated by activated macrophages and neutrophils oxidatively inactivate lysyl oxidase and other crosslink-stabilizing enzymes, shifting the equilibrium toward net crosslink loss [41,42]. This oxidative mechanism acts synergistically with protease-mediated ECM degradation to impair network integrity at two levels simultaneously: reducing crosslink formation and accelerating crosslink removal [43,44].

### 2.3. Cellular Dynamics: Repair, Inflammation, and Destruction

Lung structural homeostasis is actively maintained by a population of resident and recruited cells. Fibroblasts are the principal producers of ECM components, synthesizing procollagen and tropoelastin precursors and secreting lysyl oxidase [45,46]. In their reparative phenotype, fibroblasts respond to mechanical stress and cytokine signals by upregulating ECM synthesis and remodeling activity. However, in the setting of chronic injury, fibroblasts may undergo phenotypic transitions, involving reduced synthetic activity or activation states that paradoxically favor matrix degradation [47,48].

Macrophages occupy a central role in regulating the activity of fibroblasts and immune cells in the cooperation–defection process. Alveolar macrophages of the M2 phenotype are anti-inflammatory, promoting tissue repair and fibroblast recruitment [49,50]. M1 macrophages, polarized by bacterial products or interferon-gamma, are pro-inflammatory and secrete large quantities of MMPs, reactive oxygen species, and cytokines that recruit and activate neutrophils [50,51]. In chronic obstructive pulmonary disease (COPD), macrophage numbers are markedly elevated, and their phenotype is skewed toward the M1 pole, with increased secretion of MMP-9, MMP-12, and CXCL8 [52].

Neutrophils, in turn, are among the most potent producers of proteolytic enzymes, including neutrophil elastase, MMP-8, and MMP-9, as well as myeloperoxidase, which generates hypochlorous acid [53]. The net effect of sustained M1 macrophage and neutrophil activity is a profound elevation of proteolytic and oxidative stress that overwhelms the lung’s antiprotease defenses, particularly alpha-1-antitrypsin and secretory leukocyte protease inhibitor, and degrades ECM integrity [54]. Critically, the balance between reparative and destructive cellular phenotypes is not static: it responds dynamically to signals from the ECM itself, creating the bidirectional coupling central to our model [55].

## 3. The Game-Theoretic Framework

### 3.1. The Prisoner’s Dilemma as a Model of Structural Cooperation

The Prisoner’s Dilemma (PD) is the canonical model of cooperation under conflicting incentives [39,40,51]. In its simplest form, two agents each choose simultaneously to cooperate (C) or defect (D). If both cooperate, both receive reward R. If both defect, both receive punishment P. If one cooperates and one defects, the cooperator receives the sucker’s payoff S and the defector receives the temptation payoff T, with T > R > P > S. The defining feature of the PD is that defection is a strictly dominant strategy for each agent individually, yet mutual defection is less effective (termed Pareto-inferior) than a strategy that avoids making any agent worse off [56].

We apply the PD metaphor to ECM crosslinks by interpreting each crosslink as an agent whose strategy corresponds to its mechanical behavior under mechanical load. A cooperating crosslink remains intact, sharing the mechanical load with its neighbors and maintaining the integrity of the network. A defecting crosslink ruptures under load, transferring stress to its neighbors and increasing the probability that they, in turn, will rupture. This is not a conscious strategic choice; rather, it reflects the thermodynamic and mechanical properties of the crosslink in its local environment, which are influenced by the degree of oxidative modification, the local density of intact neighbors, and the magnitude of applied stress [57].

In the iterated version of the game, each crosslink’s strategy is updated probabilistically at each time step as a function of the current stress it experiences and its oxidative modification state. The fraction of cooperating crosslinks in the network evolves according to replicator dynamics modulated by mechanical load and by the proteolytic environment generated by cellular players. Spatial heterogeneity of the crosslink network is incorporated by embedding the game on a lattice structure, which allows cooperating and defecting clusters to form and propagate in a manner analogous to spatial evolutionary game dynamics [58,59].

### 3.2. The Cellular Game: Repair Versus Destruction

At the cellular level, we model the strategic interaction among fibroblasts, macrophages, and neutrophils using a multiplayer game in which each cell population is characterized by a phenotypic strategy ranging from cooperative (repair-oriented) to defective (damage-promoting) [46,47,50]. The payoff to a given phenotype is a function of the phenotypic composition of the local cellular community and the mechanical state of the ECM. This game structure is analogous to the multiplayer public goods game studied extensively in evolutionary game theory, in which contributors to a shared resource receive payoffs that depend on the group’s total contribution [Table 1] [60].

**Figure 4:**
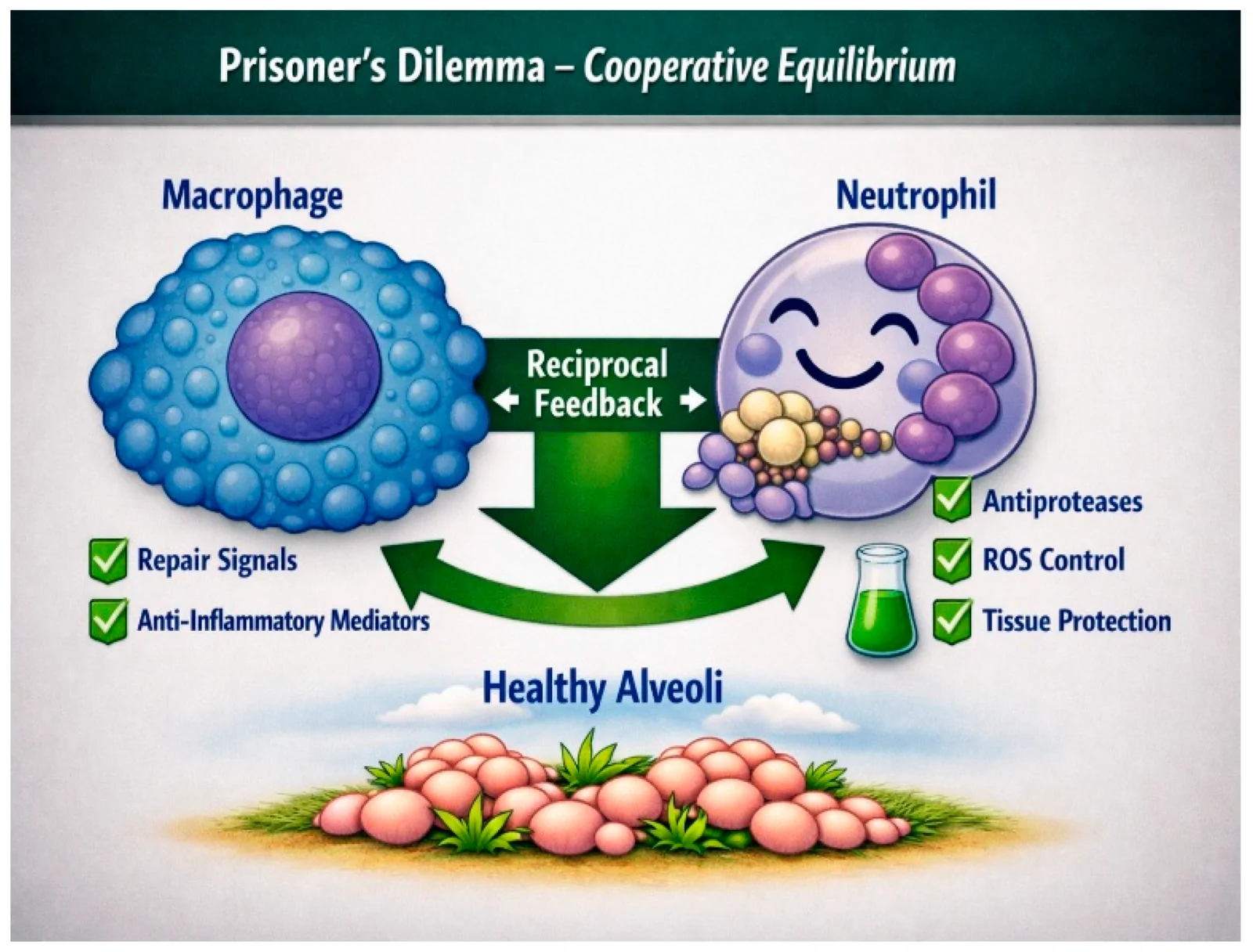
Cartoon showing a tissue environment dominated by cooperative phenotypes, where growth factors and cytokines favor phenotypic stability, mechanical cues from intact ECM reinforce reparative signaling, and the anti-inflammatory milieu suppresses transition to destructive phenotypes. (Conceptual schematic; not model output).

In a tissue environment dominated by cooperative (M2 macrophage, reparative fibroblast) phenotypes and structurally intact ECM, cooperative cells receive the highest fitness payoff, where growth factors and cytokines favor their proliferation and phenotypic stability, mechanical cues from intact ECM reinforce reparative signaling, and the anti-inflammatory milieu, rich in resolving lipid mediators such as lipoxins and resolvins, suppresses transition to destructive phenotypes [61,62,63]. Defective phenotypes (M1 macrophages, activated neutrophils) in the same environment receive a lower payoff: their inflammatory output is partially neutralized by antiprotease activity, and ECM targets of their proteases are relatively resistant to degradation [Figure 4] [64,65].

**Figure 5:**
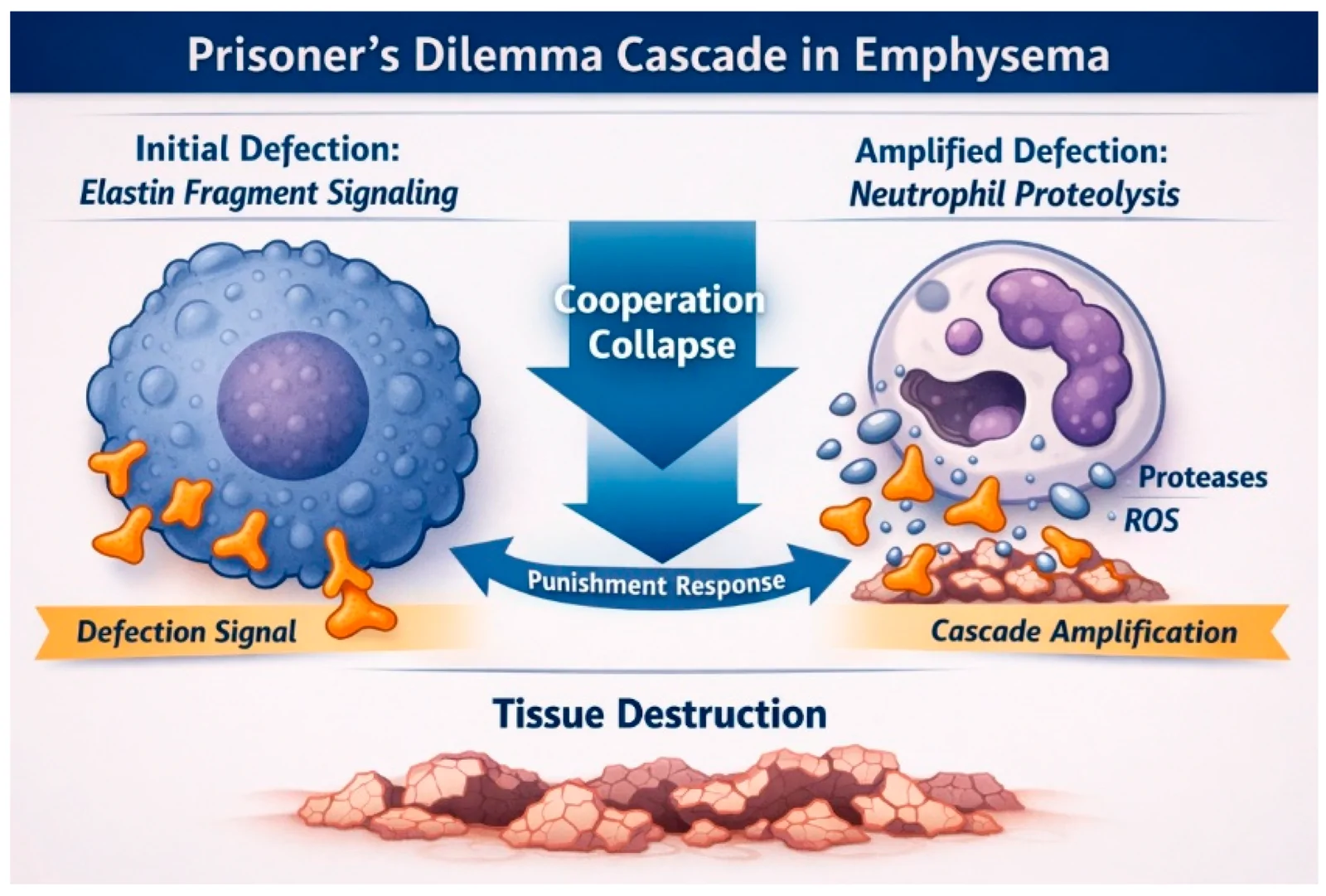
Cartoon showing a tissue environment in which degraded ECM activates toll-like receptors and RAGE on macrophages, driving M2-to-M1 polarization. The resulting inflammatory milieu suppresses fibroblast synthetic activity and promotes recruitment of neutrophils which release proteases and ROS. (Conceptual schematic; not model output).

As ECM damage accumulates, however, the payoff landscape shifts. Matrikines and DAMPs released by degraded ECM activate toll-like receptors and RAGE on macrophages, driving M2-to-M1 polarization [66,67]. The resulting inflammatory milieu suppresses fibroblast synthetic activity and promotes neutrophil recruitment. Crucially, as the cooperative cellular population shrinks, the marginal benefit of remaining cooperative falls while the marginal benefit of defecting rises, a classic cascade toward a low-cooperation equilibrium [Figure 5].

### 3.3. Bidirectional Coupling and Feedback

The defining feature of our framework is the bidirectional coupling between the ECM game and the cellular game. We formalize this as mutual payoff modulation, where the payoff matrices of both games include terms that depend on the other game’s current state [68]. The cooperation payoff in the ECM game, R^ECM^, decreases as the fraction of defective cellular phenotypes increases (reflecting elevated proteolytic and oxidative stress). Conversely, the cooperation payoff in the cellular game, R^cell^, decreases as the fraction of intact crosslinks falls below a threshold (reflecting the loss of structural ECM cues that support reparative phenotypes).

This positive feedback loop between ECM damage and cellular defection has been empirically documented in several contexts. Studies of cigarette smoke-exposed murine lungs show that macrophage MMP-12 expression rises sharply after a threshold level of alveolar wall damage is reached, suggesting a switch-like rather than a proportional response [69]. Similarly, in vitro studies demonstrate that fibroblast synthetic phenotype is acutely sensitive to substrate stiffness: fibroblasts on mechanically degraded ECM substrates downregulate collagen synthesis and upregulate MMP expression, a phenotypic switch consistent with defection in the cellular game [70].

**Figure 6:**
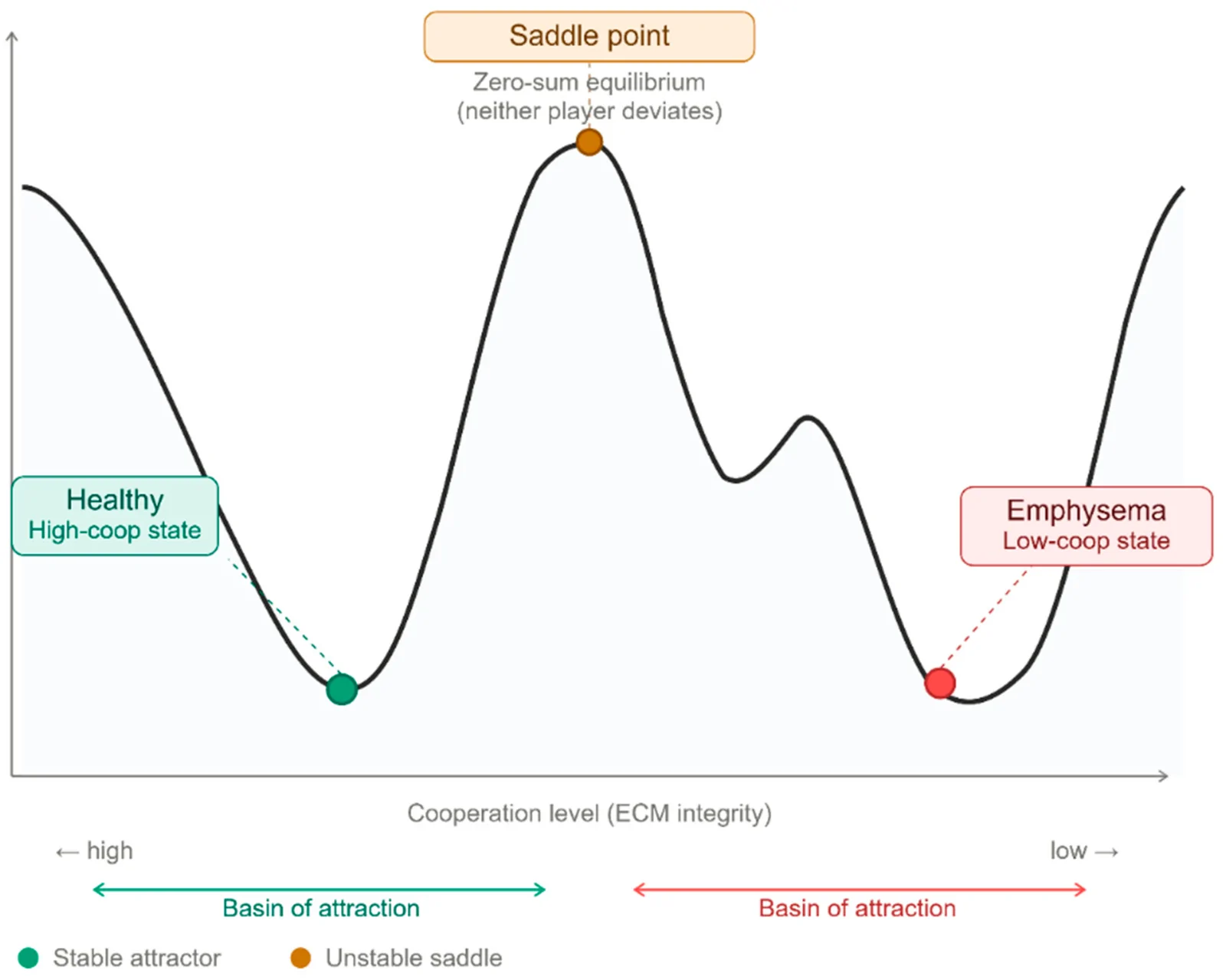
Schematic potential energy landscape as a function of cooperation level (ECM integrity). Two stable attractors (local minima) correspond to a high-cooperation “healthy” state (left) and a low-cooperation “emphysema” state (right), each with its own basin of attraction. These states are separated by an unstable saddle point representing a zero-sum equilibrium, such that perturbations determine transitions between the two regimes.

The coupled dynamical system exhibits bistability, where two stable fixed points are separated by an equilibrium in a zero-sum game. This condition, termed a saddle point, occurs when a player’s maximized gain aligns with the opponent’s minimum loss, and neither player has an incentive to unilaterally change their strategy. This equilibrium may be associated with a high-cooperation state, corresponding to healthy tissue, or a low-cooperation state, corresponding to established pulmonary emphysema [Figure 6].

### 3.4. Formal Model Specification, Parameter Sensitivity, and Model Comparison

To formalize the coupled ECM–cellular dynamics described above, we track two quantities that summarize the cooperative behavior of the lung: x(t), the fraction of intact elastin and collagen crosslinks, and y(t), the fraction of reparative (cooperative) fibroblasts, macrophages, and neutrophils. Both variables range from 0 to 1. The essential feature of the model is that each subsystem directly influences the other. When cellular phenotypes shift toward destructive behavior, they release proteases and oxidants that reduce the stability of ECM crosslinks. Conversely, when the ECM becomes damaged, it generates biochemical danger signals that push macrophages and fibroblasts toward inflammatory, matrix-degrading phenotypes. This creates a feedback loop in which damage in one system encourages damage in the other.

We represent this interaction by allowing the payoff for cooperation in each subsystem to depend on the state of the other. The cooperation payoff for ECM crosslinks is written as

$$R_{\mathrm{ECM}}\left(y\right)=R_{0,\;\mathrm{ECM}}-\alpha \left(1-y\right),$$

where α measures how strongly destructive cellular phenotypes reduce the reward for maintaining crosslink integrity. Similarly, the cooperation payoff for reparative cellular phenotypes is

$$R_{\mathrm{cell}}\left(x\right)=R_{0,\;\mathrm{cell}}-\beta \left(1-x\right),$$

where β measures how strongly loss of ECM integrity reduces the reward for maintaining reparative cellular behavior. These payoff functions enter standard replicator equations describing how the fraction of cooperators changes over time:

$$dx/dt=x(1-x)[\pi_{C}(x,y)-\pi_{D}(x,y)],$$

$$dy/dt=y(1-y)[\pi_{C}\;(y,x)-\pi_{D}(y,x)],$$

where $\pi_{C}$ and $\pi_{D}$ are the expected payoffs to cooperation and defection, computed from the payoff structure in Table 1. Because both cooperation payoffs decrease as the opposing population defects, the two equations are positively coupled: decreases in *y* reduce the incentive for ECM cooperation, and decreases in *x* reduce the incentive for cellular cooperation. This coupling is the mathematical engine driving runaway collapse once the system approaches its tipping point.

Solving dx/dt=dy/dt=0 yields the system’s fixed points. When the feedback strength—quantified by the product αβ—is small, the system has a single stable equilibrium corresponding to healthy lung tissue. When αβ exceeds a critical threshold determined by the temptation–reward gap (T − R), the system develops three fixed points: a stable high-cooperation state (healthy), a stable low-cooperation state (emphysema), and an intermediate saddle point that acts as a tipping point. Once the system crosses this saddle, even slightly, it will continue drifting toward emphysema even if the original injurious stimulus is removed. This mechanism explains the clinical observation that emphysema often progresses slowly for years and then accelerates abruptly.

The ECM variable **x** also maps directly onto the intact-bond probability p in percolation theory. Near the percolation threshold $p_{c}$, the size of the mechanically effective giant component changes sharply with small changes in **x**. Thus, gradual biological drift in crosslink integrity can produce sudden mechanical failure once the network approaches its connectivity threshold. Human postmortem data showing a sharp rise in elastin breakdown once alveolar diameter reaches ~400 µm are consistent with this predicted threshold behavior.

A sensitivity analysis shows that the location of the tipping point is most strongly influenced by α (protease/oxidant sensitivity of ECM cooperation), β (dependence of cellular phenotype on ECM integrity), and the percolation threshold $p_{c}$. Baseline cooperation rewards $R_{0,\mathrm{ECM}}$ and $R_{0,\mathrm{cell}}$ exert comparatively weaker influence. This leads to a practical prediction: therapies that protect crosslinks or strengthen ECM connectivity should be more effective at preventing collapse than therapies that simply increase repair signals. Finally, the model predicts that both the exponential rise in free desmosine and the sigmoidal rise in total desmosine density should share the same inflection point, because both processes are governed by the same underlying threshold in ECM connectivity. This is precisely what the human data show. Alternative models can fit each curve separately but do not explain why the two curves bend at the same point, giving the coupled ECM–cellular model greater explanatory power.

## 4. Percolation Theory and the Tipping Point

### 4.1. Network Connectivity and Critical Thresholds

**Figure 7:**
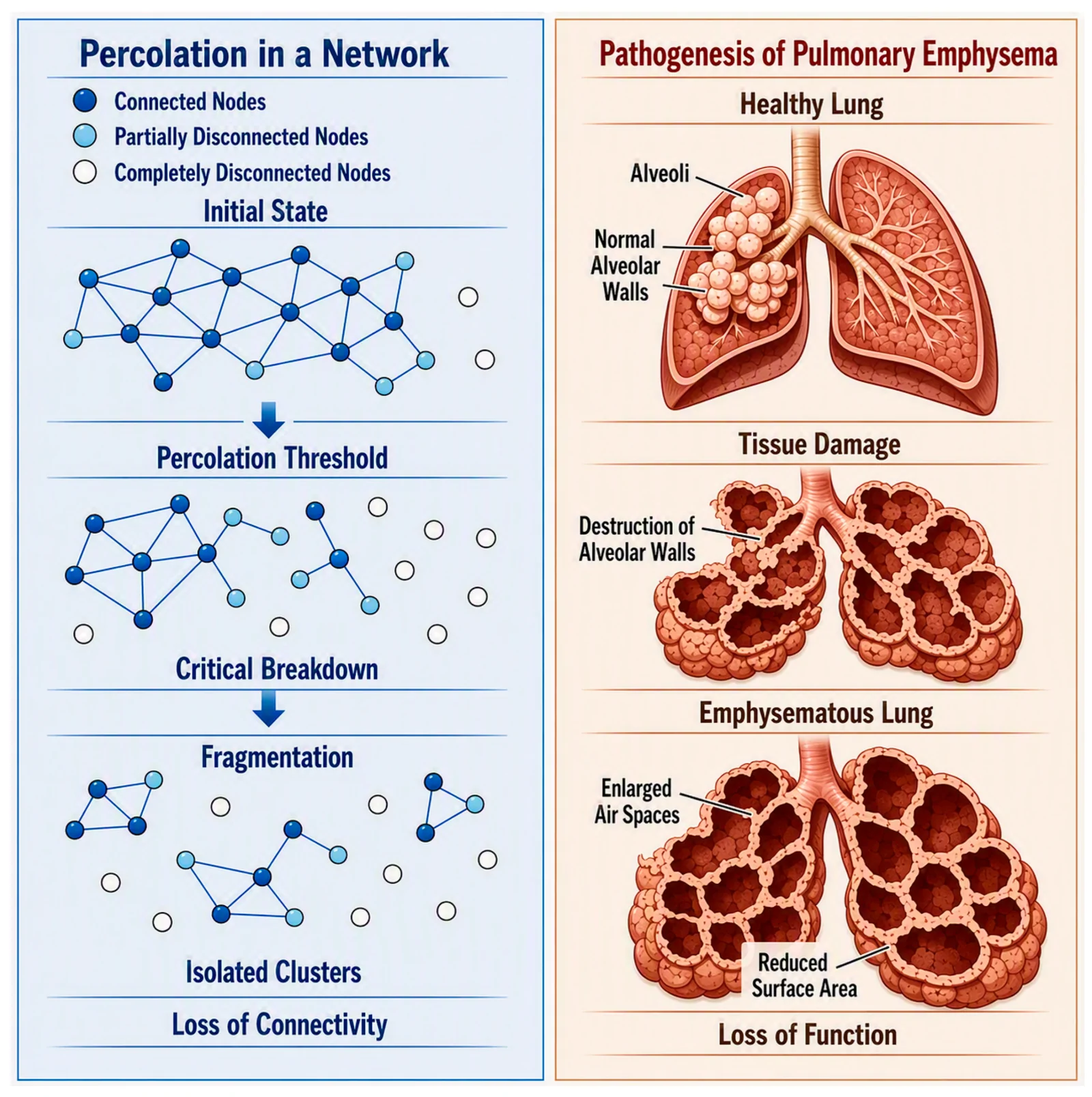
As applied to the lung, percolation theory proposes the existence of a critical threshold p(c), such that for p > p(c) the collagen and elastin network contains a spanning connected cluster capable of transmitting mechanical load across the tissue, while for p < p(c), the network fragments into small, disconnected clusters and loses this capacity. (Conceptual schematic; not model output).

The connection between our game-theoretic model and observable tissue destruction is mediated by percolation theory. We model the ECM crosslink network as a random graph in which each bond is independently intact with probability p. Percolation theory establishes that for a broad class of network topologies, there exists a critical threshold p(c) such that for p > p(c) the network contains a spanning connected cluster capable of transmitting mechanical load across the tissue, while for p < p(c), the network fragments into small, disconnected clusters and loses this capacity [Figure 7] [71].

The percolation transition is sharp: the size of the giant component decreases continuously to zero as p approaches p(c) from above, but the transition from a mechanically competent to a mechanically incompetent state occurs over a narrow range of p [72]. In the context of emphysema, this means that tissue can sustain substantial crosslink loss with only modest impairment of mechanical function, consistent with the observation that substantial emphysema may be radiographically apparent before significant spirometric decline, but that once p falls below p(c), mechanical function deteriorates precipitously.

Near the percolation threshold, the distribution of local stress concentrations becomes extremely heterogeneous: most crosslinks bear very little load, but a small fraction near defects in the network bear stress far above the network average [73]. These highly stressed crosslinks are prone to oxidative damage and mechanical fatigue, creating a local instability that preferentially propagates failure. This mechanism explains the spatial heterogeneity of airspace enlargement in pulmonary emphysema as a consequence of local variations in initial crosslink density and oxidative load that are amplified by the cooperative nature of failure propagation [74].

The 400 µm MLI threshold identified by Fagiola et al. in human emphysematous tissue provides compelling empirical support for a percolation-like tipping point in the ECM crosslink game [8]. Below this diameter, free DID levels remain low, and DID density rises sigmoidally, consistent with a tissue still in the high-cooperation basin of attraction, actively remodeling elastin to counteract mounting mechanical strain. Above 400 µm, free DID increases exponentially while DID density plateaus, indicating that the repair mechanism has been overwhelmed and that the balance has shifted irreversibly toward net elastin destruction. This inflection corresponds precisely to the saddle point in the bistable dynamical system. It is the alveolar size at which the percolation network loses sufficient connectivity to distribute mechanical load cooperatively, local stress concentrations exceed the tensile capacity of individual crosslinks, and defection cascades propagate faster than repair can compensate. The sigmoid compensatory phase and the subsequent exponential breakdown phase thus map directly onto the pre-threshold and post-threshold regimes of the game-percolation model, and the 400 µm boundary represents an empirically measurable approximation of p(c) in human lung tissue.

### 4.2. Irreversibility as a Feature of Percolation Dynamics

A critical feature of pulmonary emphysema is its irreversibility. Once significant alveolar destruction has occurred, cessation of smoking or other interventions do not restore lung architecture [75]. Our framework provides a mechanistic explanation for this irreversibility in terms of hysteresis in the coupled game-percolation system. Because the system is bistable, once the state trajectory crosses the tipping threshold and is captured by the low-p attractor, returning to the healthy attractor requires not merely removing the perturbation but actively pushing the system back across the tipping threshold in the opposing direction.

In practical terms, this means that restoring crosslink integrity requires not only the cessation of protease- and oxidant-mediated crosslink destruction but also the active regeneration of new crosslinks at a rate sufficient to push p back above p(c). The ECM in emphysematous lung, however, presents a hostile environment for this regeneration. Degraded ECM cannot provide the structural scaffold necessary for organized fibroblast activity, the inflammatory milieu suppresses fibroblast synthetic phenotypes, and the absence of mechanical tension in collapsed alveolar walls fails to provide the mechanotransductive signals that normally stimulate collagen and elastin synthesis [76]. The system is thus trapped in a self-maintaining low-cooperation equilibrium even in the absence of ongoing exogenous injury.

Formally, this hysteresis follows from the saddle-node bifurcation structure derived in Section 3.4 because the return trajectory must re-cross the same saddle point from the opposite side, Furthermore, conditions within the low-cooperation attractor act to increase, rather than decrease, the composite bifurcation parameter that governs bistability. Consequently, spontaneous reversal is dynamically forbidden without an external intervention that changes system parameters, not merely initial conditions.

## 5. Explaining the Characteristic Features of Pulmonary Emphysema

### 5.1. Latency and Sudden Acceleration

One of the most puzzling aspects of pulmonary emphysema in smokers is its long preclinical latency. Most individuals who develop the disease smoke for decades before a significant spirometric decline is detected, and many heavy smokers never develop clinically significant disease [77]. Our framework precisely predicts this behavior. When the coupled game system is initialized at the high-cooperation attractor, small perturbations produced by cigarette smoke exposure cause the state to oscillate near the attractor without crossing the tipping threshold because, at high p, the network’s cooperating crosslinks can distribute mechanical stress effectively, and reparative cellular phenotypes can locally restore damaged ECM.

However, as cumulative oxidative and proteolytic damage accumulates over years of exposure, the basin of attraction of the healthy state gradually shrinks. Whether a particularly large inflammatory episode pushes the state across the critical threshold, or the slow drift in baseline phenotypic composition modifies this threshold, the disease trajectory crosses the saddle point and enters the basin of the destroyed-tissue attractor. From the perspective of an external observer measuring FEV_1_, this transition appears as a sudden acceleration of decline following a prolonged period of apparent stability, a pattern documented in longitudinal studies of COPD progression [78].

This tipping-point mechanism also explains the high variability in susceptibility to emphysema. Individuals differ in their initial ECM crosslink density, baseline antiprotease capacity, macrophage polarization thresholds, and antioxidant enzyme expression, all of which determine how large a perturbation is required to cross the tipping threshold [79]. The high percentage of heavy smokers who never develop significant emphysema corresponds, in this framework, to individuals whose system parameters maintain a wide basin of attraction for the healthy state throughout their lifetime.

### 5.2. Spatial Heterogeneity

**Figure 8:**
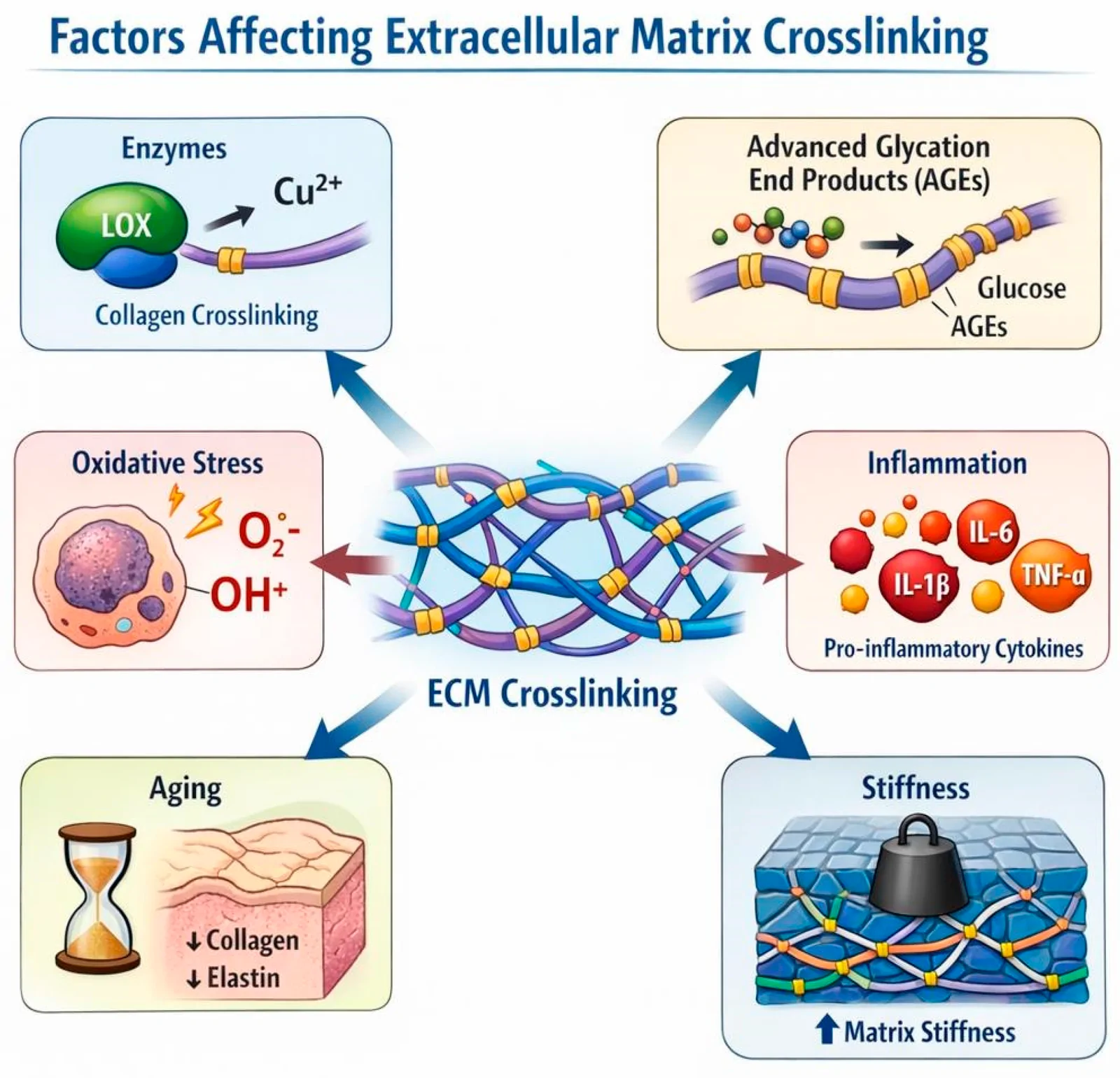
The crosslinking density in the lung ECM is based on a number of factors whose effects may be localized, which may explain the spatial heterogeneity of pulmonary emphysema. (Conceptual schematic; not model output).

The spatial heterogeneity of emphysema, the coexistence of severely destroyed and relatively preserved regions within the same lung, is a hallmark feature that has resisted simple molecular explanation [80]. In our framework, spatial heterogeneity arises naturally from the interplay of local stochastic variation and the nonlinear dynamics of the coupled system. Because the initial distribution of crosslink density and oxidative modification is spatially nonuniform, reflecting local variations in airflow-mediated smoke deposition, regional differences in antioxidant enzyme expression, and heterogeneity in tissue mechanics, different regions of the lung may begin in different positions relative to their local tipping threshold [Figure 8].

As global inflammation increases, regions with initially lower p cross their tipping thresholds first, entering a cascade of defection that further increases local inflammation and protease secretion. This local defection cascade can propagate laterally by elevating oxidative stress in neighboring tissue and recruiting additional inflammatory cells, but the propagation rate is limited by tissue geometry and the diffusion of inflammatory mediators [81,82]. Consequently, the lung can exist in a state of spatial phase coexistence, with some regions in the destroyed-tissue attractor and others still in the healthy attractor, a pattern precisely mirrored in the radiographic and pathological appearance of centrilobular and panlobular emphysema [83].

### 5.3. Exacerbations as Perturbations near the Tipping Threshold

Acute exacerbations of COPD, episodes of worsening respiratory symptoms triggered by infection or environmental exposures, are associated with accelerated long-term lung function decline that cannot be fully explained by the acute insult alone [84,85]. In our framework, exacerbations represent large transient perturbations to the coupled game system. In a patient whose system state is far from the tipping threshold, the system recovers after the exacerbation resolves and returns to its pre-exacerbation basin of attraction. In a patient whose state is close to the threshold, a sufficiently large exacerbation may push the trajectory across the saddle point into the destroyed tissue attractor. Recovery is then impossible without active intervention, manifesting clinically as incomplete post-exacerbation recovery of FEV_1_ or the “step-down” pattern of lung function decline observed in longitudinal COPD cohorts [86].

## 6. Therapeutic Implications and Leverage Points

### 6.1. Stabilizing Cooperation: Early Intervention

The most important practical implication of the bistable, threshold-crossing model is that therapeutic intervention is far more efficacious before the tipping threshold is crossed than afterward. In the early phase of disease, when the system is still in the basin of the healthy attractor, interventions that reduce the rate of crosslink defection or increase the frequency of cellular cooperation can substantially enlarge the basin of attraction and raise the threshold, effectively delaying or preventing the transition to irreversible disease. This provides a rigorous theoretical basis for aggressive early intervention, an approach supported empirically by data showing that smoking cessation is dramatically more effective at preserving lung function when implemented early in the disease course [87,88].

Within the game-theoretic framework, stabilizing cooperation at the cellular level is equivalent to shifting payoff matrices to increase R^cell^ and decrease the temptation payoff for defection. Candidate interventions include alpha-1-antitrypsin augmentation therapy for individuals with inherited deficiency, broad-spectrum MMP inhibitors, suppression of M1 macrophage polarization with phosphodiesterase-4 inhibitors, and enhancement of the fibroblast reparative phenotype through TGF-beta pathway modulation.

### 6.2. The Percolation Threshold as a Therapeutic Target

The percolation threshold p(c) represents a specific and quantifiable target for therapy: any intervention that maintains p above p(c), even in the presence of ongoing crosslink damage, will prevent the mechanical collapse of global network connectivity [89]. This observation suggests that the therapeutic goal need not be complete suppression of crosslink damage, an unrealistic objective in the context of chronic inflammation, but rather partial protection sufficient to keep the intact crosslink fraction above the critical threshold.

Importantly, the value of p(c) depends on the topology of the crosslink network, which is itself modifiable. In a network with higher average connectivity, where p(c) is lower, more bonds can be lost before global connectivity fails. This suggests that interventions aimed at increasing the branching and redundancy of the ECM crosslink network, such as promoting collagen fibril branching or increasing crosslink density per fiber, could raise the network’s robustness to damage. Lysyl oxidase-like proteins (LOXL2, LOXL4), which catalyze specific types of crosslink formation, are potential molecular targets for such interventions [90].

### 6.3. Reversing Cellular Defection: The Challenge of Resetting Payoffs

In established pulmonary emphysema, the cellular game is locked in the defection attractor, where the local ECM environment generates persistent matrikine signaling that maintains M1 macrophage polarization and neutrophil recruitment, creating a self-sustaining inflammatory cascade even in the absence of exogenous stimuli. Breaking this cycle requires not merely reducing inflammatory inputs but actively resetting the payoff landscape of the cellular game by restoring conditions under which cooperative phenotypes have higher fitness than destructive ones.

Candidate strategies for resetting cellular payoffs include the use of resolving mediators of inflammation such as lipoxins, resolvins, and protectin D1, which actively promote M2 macrophage polarization and neutrophil apoptosis, thereby reducing the defecting cellular fraction [91]. Delivery of decellularized ECM scaffolds or bioengineered matrix hydrogels into emphysematous regions could provide structural cues that favor the fibroblast reparative phenotype and locally increase R^cell^, potentially drawing the local cellular game back toward the cooperative attractor [92,93]. Lung-volume reduction surgery, which restores mechanical tension to chronically over-distended emphysematous tissue, may also shift mechanotransductive signaling toward a more cooperative cellular phenotype, providing a mechanistic interpretation of its documented functional benefit [94].

Cell-based therapies using mesenchymal stem cells, which natively secrete paracrine factors that promote M2 polarization and ECM repair, represent another approach to resetting the payoff environment in favor of cooperation [95]. Pharmacological activation of specialized reparative pathways with receptor agonists selective for lipoxin A4 receptors or resolvin receptors may provide a more targeted means of inducing phenotypic transition in macrophages without the off-target effects of broad immunosuppression [96].

The framework’s most distinctive therapeutic implication, relative to conventional single-target therapy, is that it identifies threshold-proximity biomarkers (Section 8) as a rationale for combination therapy. The pairing of a percolation-threshold-stabilizing intervention (Section 6.2) with a payoff-resetting intervention (Section 6.3) should be synergistic, specifically when the system is near the tipping point, in a way that neither intervention alone (or either intervention given well before or after threshold-crossing) would reproduce. This is a quantitatively specific, falsifiable prediction that is not generated by non-game-theoretic, single-target models of emphysema therapy, and it suggests that biomarker-guided timing of combination therapy, rather than the specific agents used, may be the primary lever this framework adds to existing therapeutic strategy.

## 7. Application to the Pathogenesis of Combined Pulmonary Fibrosis and Emphysema

### 7.1. The Clinical Puzzle of CPFE

**Figure 9:**
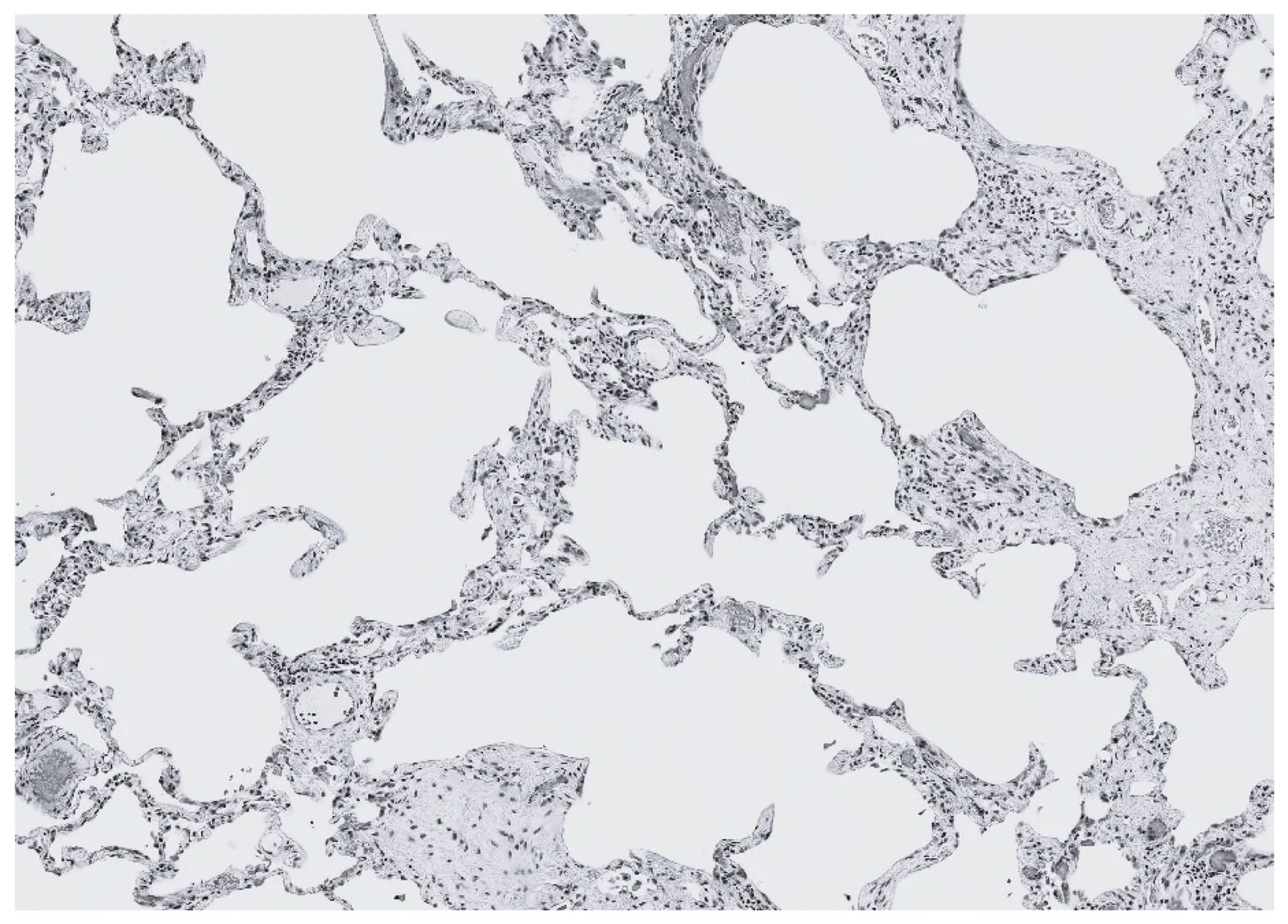
Photomicrograph of a human lung showing enlarged airspaces and interstitial fibrosis, consistent with CPFE.

Combined pulmonary fibrosis and emphysema (CPFE) is a syndrome, identified predominantly in heavy smokers, in which upper-lobe-predominant emphysema coexists with lower-lobe-predominant pulmonary fibrosis within the same lung [Figure 9] [97,98]. Patients with CPFE present a functional profile that defies the expectations of either disease occurring alone, where spirometric indices such as FEV_1_, FVC, and total lung capacity are often relatively preserved or even normal, while the diffusing capacity for carbon monoxide (DLCO) is severely reduced [99,100]. Pulmonary arterial hypertension develops in roughly half of patients and is associated with a markedly worse prognosis than either pure emphysema or pure idiopathic pulmonary fibrosis [101]. The juxtaposition within a single organ, often within centimeters, of net crosslink loss with airspace enlargement and net crosslink gain with airspace obliteration, represents a stringent test of any unifying model of ECM–cellular pathobiology. To help resolve this paradox, the multiscale game-theoretic framework introduced above is extended to include an additional strategy that accommodates the unique features of CPFE.

It is emphasized at the outset that the extension developed in this section is offered as a hypothesis-generating application of the core framework to CPFE, and not as an established mechanism. The three-strategy game, the dual-threshold percolation model, and the associated molecular predictions below, including the proposed roles of the MUC5B promoter polymorphism and telomerase mutations in biasing regional attractor selection (Section 7.5), should be read as testable hypotheses generated by the framework rather than as conclusions derived from it.

### 7.2. A Third Cellular Strategy: Pathological Hypersynthesis

The cellular game described in Section 3.2 was framed as a binary contest between reparative, cooperative phenotypes and protease- or oxidant-secreting, defective phenotypes, a framing sufficient to capture pure emphysema, in which the destructive attractor is characterized by net matrix degradation. Idiopathic pulmonary fibrosis, and the fibrotic compartment of CPFE, is not characterized by an absence of fibroblast activity but by its pathological excess, where fibroblasts differentiate into myofibroblasts that secrete large quantities of crosslinked collagen, resist apoptosis, and form the fibroblastic foci that are the histological hallmark of usual interstitial pneumonia [102]. This is not simply additional cooperation in the original sense of the term, because the resulting matrix is not homeostatically regulated; it is excessive, architecturally disordered, and itself the proximate cause of functional impairment through loss of compliance and obliteration of the alveolar-capillary interface [103].

We therefore extend the cellular game from a binary to a three-strategy contest, adding a hypersynthetic strategy (H) alongside cooperation (C) and defection (D). A cell adopting H continues to secrete ECM components and lysyl oxidase, in this sense resembling C, but does so in a manner uncoupled from the mechanical and biochemical feedback that normally limits matrix deposition to the amount required for structural maintenance. The payoff to H, unlike the payoff to C, is enhanced rather than suppressed by signals associated with epithelial injury, including senescence-associated secretory phenotype factors, transforming growth factor-beta, and Wnt ligands released by chronically injured or senescent alveolar epithelial cells [104]. Whereas the cooperative-to-defective transition described in Section 3.2 is driven primarily by accumulating proteolytic and oxidative burden acting on macrophages and neutrophils, the cooperative-to-hypersynthetic transition is driven by a distinct upstream signal involving repetitive epithelial micro-injury and senescence, transduced through an epithelial signaling layer that sits above the fibroblast game in a manner analogous to the role macrophages and neutrophils play in the original model.

### 7.3. Divergent Crosslink Games: A Dual-Threshold Percolation Model

At the ECM level, the consequence of sustained hypersynthesis is not the percolation collapse described in Section 4 but its mechanical mirror image. Excess, disordered crosslinking increases local stiffness, reduces compliance, and progressively obliterates alveolar architecture even as the crosslink network itself becomes denser and, in a strict graph-theoretic sense, more connected. A complete percolation account of ECM-driven lung disease therefore requires two critical thresholds rather than one: a lower threshold, p(c), below which the network fragments and loses load-bearing capacity (the emphysema attractor described in Section 4.1), and an upper threshold, p(c)*, above which excessive crosslink density and disordered fiber geometry produce a rigid, noncompliant network that is itself incompatible with normal alveolar mechanics and gas transfer (the fibrotic attractor). Healthy lung tissue occupies the intermediate window between p(c) and p(c)*, in which crosslink turnover is balanced, and network topology preserves both mechanical competence and the fine geometric architecture required for gas exchange.

This dual-threshold structure offers a potential explanation for the distinctive physiology of CPFE. In the emphysematous compartment, p falls below p(c), producing the obstructive, hypercompliant physiology of conventional emphysema; in the fibrotic compartment, p rises above p(c)*, producing the restrictive, hypocompliant physiology of conventional fibrosis. Because spirometric volumes are sensitive to the balance of these opposing mechanical regimes, the two processes can partially cancel at the level of FEV1, FVC, and total lung capacity, producing the deceptively preserved spirometry that is a defining and clinically treacherous feature of CPFE [105]. DLCO, by contrast, is determined by the total area and integrity of the functioning alveolar-capillary membrane and is degraded by both attractors simultaneously, airspace destruction in emphysematous regions and capillary obliteration and membrane thickening in fibrotic regions, consistent with the disproportionate reduction in DLCO observed clinically [106].

### 7.4. Cross-Attractor Coupling at the Fibrosis–Emphysema Interface

**Figure 10:**
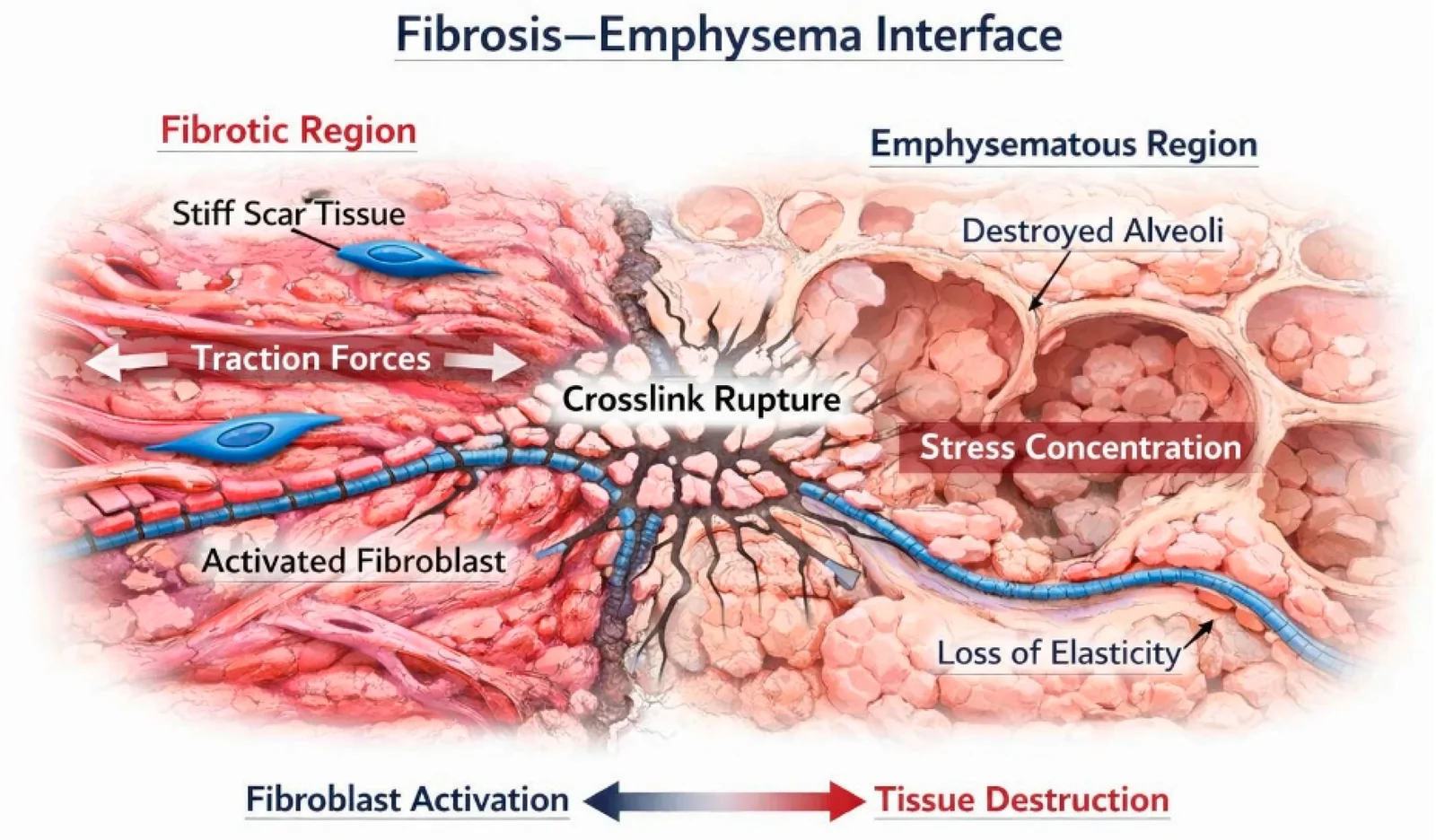
The juxtaposition of fibrotic and emphysematous regions in the lung provides a bidirectional, cross-attractor coupling that is mutually reinforcing. This mechanism may explain why CPFE carries a worse prognosis than either emphysema or fibrosis alone. (Conceptual schematic; not model output).

Section 5.2 described how spatial heterogeneity in pulmonary emphysema arises from regional variation in the position of p relative to a single tipping threshold, producing coexisting healthy and destroyed regions within one organ; CPFE requires a further extension to coexisting regions governed by two distinct pathological attractors that are mechanically coupled to one another. Fibrotic regions, by virtue of their increased stiffness, exert traction on adjacent, more compliant parenchyma, concentrating mechanical stress at the fibrosis–emphysema interface. Within the framework developed in Section 4.1, locally elevated stress increases the probability of crosslink rupture in the adjacent, lower-density network, predicting an accelerated local approach to p(c) and favoring emphysematous progression specifically at the margins of fibrotic foci. Conversely, loss of mechanical tethering in adjacent emphysematous regions removes a source of physiological mechanotransductive signaling that would otherwise restrain fibroblast activation, potentially permitting unchecked progression of the hypersynthetic attractor at the fibrotic margin. This bidirectional, cross-attractor coupling, each pathological compartment destabilizing its neighbor, offers a candidate mechanism for the observation that CPFE carries a worse prognosis than either emphysema or fibrosis alone, since the two attractors are not merely co-located but mutually reinforcing [Figure 10].

### 7.5. Determinants of Regional Attractor Selection

A central question raised by this extension is what determines, in a given smoker, whether a given region of lung evolves toward the low-p (emphysematous) attractor, the high-p (fibrotic) attractor, or remains within the healthy intermediate window. Within the model, this is governed by the relative magnitude of two upstream payoff-modulating signals: proteolytic and oxidative burden acting through macrophages and neutrophils, versus epithelial injury and senescence signaling acting through the proposed epithelial–fibroblast layer. Genetic and molecular variation that biases this balance offers a natural account of why some heavy smokers develop pure emphysema, others develop pulmonary fibrosis, and a subset develop the regionally mixed CPFE phenotype. The MUC5B promoter polymorphism, strongly associated with pulmonary fibrosis and thought to act through chronic low-grade epithelial injury and impaired mucociliary clearance, would be predicted within this framework to bias affected regions toward the hypersynthetic attractor [107]. Telomerase mutations, identified both in smokers with severe emphysema and in familial pulmonary fibrosis, may act on a shared upstream vulnerability of alveolar epithelial cells to senescence, providing a genetic background capable of biasing the system toward either attractor depending on additional local and environmental modifiers [108,109]. This view reframes CPFE not as the coincidental co-occurrence of two unrelated diseases but as the regionally divergent output of a single underlying coupled game system acting on a lung with spatially heterogeneous genetic and microenvironmental modifiers of attractor selection.

### 7.6. Implications for Diagnosis, Prognosis, and Therapy

The dual-threshold extension carries direct clinical implications. Because spirometry can be deceptively preserved in CPFE, the framework predicts that DLCO and cross-sectional imaging, rather than spirometry alone, are the appropriate tools for detecting and staging the disease, a conclusion that aligns with current clinical guidance for CPFE recognition [110]. The framework further predicts that the marked propensity of CPFE toward pulmonary hypertension reflects the combined, and possibly synergistic, loss of pulmonary capillary surface area from both attractors operating simultaneously within the same vascular bed, rather than from either process alone. Therapeutically, the model suggests that interventions effective in pure emphysema or pure fibrosis may need to be deployed with caution in CPFE. Strategies that aggressively suppress fibroblast activity to halt progression of the hypersynthetic attractor could remove reparative capacity needed to keep adjacent, mechanically stressed emphysematous regions above their local percolation threshold, illustrating how the cross-attractor coupling described in Section 7.4 complicates therapeutic strategy in ways not anticipated by single-disease models. Disentangling these coupled dynamics, rather than treating the fibrotic and emphysematous compartments as independent therapeutic targets, may represent the central clinical challenge posed by this syndrome.

## 8. Discussion

The framework presented here represents a substantial departure from conventional linear models of emphysema pathogenesis, in which disease progression is portrayed as a steady accumulation of molecular damage proportional to the intensity and duration of exposure. While the molecular components of that model, including protease–antiprotease imbalance, oxidative stress, and ECM degradation, are well established and fully incorporated into our framework, the game-theoretic and percolation perspective reveal that these molecular events acquire their pathological significance only in their collective, network-level context.

Several limitations of the current model warrant acknowledgment. The formal assignment of game-theoretic payoff values to crosslinks and cell populations is necessarily schematic at this stage; precise parameterization will require quantitative data on crosslink failure rates as a function of local stress and oxidative modification, as well as on phenotypic switching kinetics for the relevant cell populations. Network topology in the lung is not a simple random graph but reflects the complex three-dimensional architecture of alveolar walls and their fibrous reinforcement. Consequently, accurate percolation modeling will require realistic network representations derived from high-resolution imaging of emphysematous tissue.

Nevertheless, even at a qualitative level, the framework makes readily testable predictions. The existence of a bistable system with a tipping threshold predicts that the statistical distribution of emphysema progression rates in a population should be bimodal, with one cluster showing slow progression and another showing rapid, accelerating progression. This prediction is broadly consistent with epidemiological data on FEV_1_ decline rates from longitudinal cohort studies [111,112]. The framework also predicts that spatial patterns of emphysema should show characteristics of nucleation and propagation from initial foci, a prediction testable by serial high-resolution CT imaging analyzed with spatial statistics tools.

These predictions are not generic to any bistable threshold system. A simple bistable ODE model fit only to aggregate FEV_1_ trajectories would predict bimodal progression and a critical transition, but it carries no structural content about where in the lung that transition should occur or what its spatial signature should look like. The percolation-game framework, by contrast, predicts that the spatial pattern of tissue destruction should show measurable clustering signatures characteristic of percolation near p(c), including the following: (1) a power-law cluster-size distribution and a spanning-cluster correlation length that grows as p approaches p(c) from above; (2) a clustering coefficient of intact crosslinks that declines nonlinearly, and more steeply than a proportional-damage null model, as MLI approaches the approximately 400 µm empirical threshold; (3) the lasting effect of a given exacerbation on lung function should scale with the local proximity of the pre-exacerbation state to the tipping threshold rather than with exacerbation severity alone, a distinction testable with paired pre- and post-exacerbation imaging. The framework’s central percolation-based claim would be substantially weakened, and the model falsified in its present form, if either of the following conditions are met: (1) serial high-resolution CT or histological mapping showing tissue destruction propagating along spatially random or purely diffusion-limited gradients rather than threshold-nucleated, cluster-propagating patterns. or (2) crosslink clustering statistics tracked a simple exponential decay indistinguishable from proportional damage accumulation.

Perhaps most importantly, the framework identifies the early phase of disease, before the tipping threshold is crossed, as the critical therapeutic window. This finding reinforces the clinical importance of emphysema screening in at-risk populations, particularly current and former smokers, using spirometry and CT imaging. Biomarkers capable of reporting the current distance of the system from the tipping threshold, perhaps derived from circulating matrikine levels, ECM turnover markers, MMP/TIMP ratios, or monocyte polarization state, could serve as practical clinical guides to the urgency and intensity of intervention [113,114,115,116].

As shown in CPFE, the multiscale coupling at the heart of this model also has implications beyond pulmonary emphysema and may potentially accommodate diseases where ECM-cellular game dynamics primarily involve fibrogenesis, including idiopathic pulmonary fibrosis, osteoarthritis, and atherosclerotic plaque destabilization. In each of these conditions, the clinical enigma of sudden-onset complications following a long latency period may reflect the crossing of a percolation threshold by a slowly evolving cooperative-defective system.

The relationship between COPD exacerbation frequency and long-term disease trajectory is also naturally accommodated within this framework. Frequent exacerbations, as shown in multiple longitudinal cohorts, are associated with accelerated lung function decline independent of baseline severity [117,118]. In the bistable game framework, each exacerbation is a stochastic perturbation whose probability of causing a permanent transition scales with the proximity of the current state to the tipping threshold, explaining why exacerbations cause more lasting harm in patients with more advanced disease.

## 9. Conclusions

We have developed a multiscale game-theoretic framework for understanding emphysema progression in which ECM crosslinks and lung cell populations are modeled as strategic agents whose collective behavior determines tissue fate. The Prisoner’s Dilemma structure at both scales, coupled bidirectionally through payoff modulation, generates a dynamical system capable of explaining the most clinically challenging features of emphysema: the prolonged latency followed by sudden acceleration, the spatial heterogeneity of tissue destruction, and the irreversibility of established disease. These features emerge not from qualitative differences in molecular mechanism but from the collective nonlinear dynamics of cooperating and defecting agents in a network near its percolation threshold.

The framework offers new conceptual and practical tools for designing a therapeutic strategy. Rather than framing treatment as the suppression of individual molecular pathways, it frames treatment as the stabilization of cooperation at both the ECM and cellular scales, and the maintenance of crosslink network connectivity above the critical percolation threshold. The most powerful leverage points are those that simultaneously shift payoffs toward cooperation at multiple scales, such as anti-inflammatory interventions combined with ECM-stabilizing therapies, and are applied before the system crosses its tipping point into irreversible disease.

As quantitative data on ECM network topology, crosslink failure kinetics, and cellular phenotypic switching become increasingly available through advances in imaging, proteomics, and single-cell transcriptomics, the qualitative framework presented here can be refined into a quantitative predictive model to guide personalized therapeutic decisions. In this framework, the vision of pulmonary emphysema management shifts from reactive treatment of established destruction to proactive stabilization of a complex cooperative system, thereby preventing the emergence of airspace enlargement that may lead to respiratory failure.

## Figures and Tables

**Table 1:** Game Theory Payoff Based on Cell State.

Focal Cell Population (Player)	Local Microenvironment Strategy	Payoff *	Game Theory Interpretation	Biological Meaning	Role of Free-Riding Damage
Reparative fibroblast/M2 macrophage (cooperator)	Repair	(3, 3)	Cooperative equilibrium	Balanced repair maintains alveolar integrity	No free-riding; all agents contribute
Reparative fibroblast/M2 macrophage (cooperator)	Continued damage	(1, 4)	Exploitation (defection dominates)	Repairing cells incur cost while others drive inflammation/proteolysis	Healthy cells are exploited by damaging neighbors
Activated fibroblast/M1 macrophage-neutrophil (defector)	Repair	(4, 1)	Free-rider advantage	Damaged cells benefit from surrounding repair without contributing	Classic free-riding damage: damaged cells avoid repair cost but gain benefit
Activated fibroblast/M1 macrophage-neutrophil (defector)	Continued damage	(2, 2)	Defective Nash equilibrium	Chronic inflammation and tissue destruction (established emphysema)	Free-riding collapses system as cooperation disappears

* Payoff magnitudes are illustrative order-of-preference rankings (T > R > P > S, the defining condition of the Prisoner’s Dilemma) rather than fitted quantities. The focal cell population is a single representative reparative or destructive phenotype (fibroblast or macrophage/neutrophil); the local microenvironment strategy is the aggregate cooperative/defective composition of its neighboring cells, consistent with the multiplayer public-goods framing formalized in Section 3.4.

## Data Availability

No new data were created or analyzed in this study. Data sharing is not applicable to this article.
